# Caffeoylquinic Acid Derivatives of Purple Sweet Potato as Modulators of Mitochondrial Function in Mouse Primary Hepatocytes

**DOI:** 10.3390/molecules26020319

**Published:** 2021-01-09

**Authors:** Andrea Torres, Lilia G. Noriega, Claudia Delgadillo-Puga, Armando R. Tovar, Arturo Navarro-Ocaña

**Affiliations:** 1 Departamento de Alimentos y Biotecnología, Facultad de Química, UNAM, Ciudad de México 04529, Mexico; andrea_ta5@hotmail.com; 2 Departamento de Fisiología de la Nutrición, Instituto Nacional de Ciencias Médicas y Nutrición Salvador Zubirán, Ciudad de México 14080, Mexico; lgnoriegal@gmail.com (L.G.N.); armando.tovarp@incmnsz.mx (A.R.T.); 3 Departamento de Nutrición Animal Dr. Fernando Pérez-Gil Romo, Instituto Nacional de Ciencias Médicas y Nutrición Salvador Zubirán, Ciudad de México 14080, Mexico; claudia.delgadillop@incmnsz.mx

**Keywords:** purple sweet potato, CQA-derivatives, maximal respiration, glycolysis, hepatic steatosis

## Abstract

Owing to their antioxidant properties, caffeoylquinic acid (CQA)-derivatives could potentially improve the impaired metabolism in hepatic cells, however, their effect on mitochondrial function has not been demonstrated yet. Here, we evaluated the impact of three CQA-derivatives extracted from purple sweet potato, namely 5-CQA, 3,4- and 4,5-diCQA, on mitochondrial activity in primary hepatocytes using an extracellular flux analyzer. Notably, an increase of maximal respiration and spare respiratory capacity were observed when 5-CQA and 3,4-diCQA were added to the system indicating the improved mitochondrial function. Moreover, 3,4-diCQA was shown to considerably increase glycolytic reserve which is a measure of cell capability to respond to an energy demand through glycolysis. Conversely, 4,5-diCQA did not modify mitochondrial activity but increased glycolysis at low concentration in primary hepatocytes. All compounds tested improved cellular capacity to oxidize fatty acids. Overall, our results demonstrated the potential of test CQA-derivatives to modify mitochondrial function in hepatic cells. It is especially relevant in case of dysfunctional mitochondria in hepatocytes linked to hepatic steatosis during obesity, diabetes, and metabolic syndrome.

## 1. Introduction

Sweet potato (*Ipomoea batatas* L. (Lam)) was originally domesticated in tropical America, most probably in a region between the Yucatan Peninsula in Mexico and the Orinoco river in Venezuela, as demonstrated by morphological analysis [1]. Sweet potatoes are naturally rich in phenolic compounds which content varies according to the pulp color (yellow, white, or purple). The purple pulp sweet potato (PSP) has the highest total phenolic content (TPC) concentration [2]. Some researchers point out that caffeoylquinic acid derivatives (CQA-derivatives) comprise 70% of the total phenols while approximately 20% are made up by flavonoids [3]. Among major CQA structures found in PSP are 5-CQA, and three diCQA, namely 3,5-, 3,4-, and 4,5-diCQA [4]. These compounds have been known for their protective role against oxidative stress in human body preventing in this way many diseases [5].

The beneficial effects of CQA-derivatives have been widely studied and include hepatoprotective, antihistamine, and hypoglycemic activities, as well as inhibitory effects on free radicals and HIV replication, and antimutagenic properties [6,7,8]. Many biological functions played by these compounds have already been well described while others still require investigation and are currently under scrutiny [9]. Furthermore, the potential role of CQA-derivatives in metabolic diseases, such as obesity, diabetes, and metabolic syndrome, has been reviewed recently [10]. In these works, the effects of supplementation with 5-CQA [11] or extracts containing CQA-derivatives [12,13] were studied using animal models with diet-induced obesity or hyperglycemia. It was demonstrated a decreased body weight gain and a hypoglycemic effect induced by these compounds.

Several mechanisms have been proposed to explain the beneficial effect of CQA-derivatives. For example, activation of AMP-activated protein kinase (AMPK) [14] and modulation of peroxisome proliferator-activated receptor alpha (PPARα) [15] have been frequently associated with an improvement of mitochondrial function [16,17,18]. Regulation of mitochondrial activity represents a main target in the treatment of metabolic diseases, such as obesity, diabetes, or metabolic syndrome, with the aim to re-establish metabolic alterations. To our knowledge, the effect of CQA-derivatives on mitochondrial performance has not been evaluated yet. The use of extracellular flux analyzer technology represents an excellent and reliable tool to evaluate the effect of bioactive compounds on mitochondrial function, through an accurate and real time measurement of oxygen consumption rate (OCR) and extracellular acidification rate (ECAR) of live cells. Specifically, the measurement of OCR and ECAR in basal conditions and after the sequential administration of oligomycin, carbonyl cyanide-*p*-trifluoromethoxyphenylhydrazone (FCCP), rotenone and antimycin A, and 2-deoxyglucose, allows us to determine basal and maximal respiration, respiration associated to ATP synthase, spare respiratory capacity, proton leak, non-mitochondrial respiration and the glycolytic capacity of a cell [16]. Notably, measurements of the above parameters provide valuable insight into the mode of action of a test compound and its modulatory properties on the mitochondrial activity.

Thus, the aim of the present study was to evaluate the effects of three CQA-derivatives, namely 5-CQA, 3,4-diCQA and 4,5-diCQA, isolated from PSP on mitochondrial function in primary hepatocytes. For that purpose, the extracellular flux analyzer technology was used to determine whether these compounds have a potential to improve impaired mitochondrial function, which is a common characteristic of hepatic steatosis during obesity, diabetes, and metabolic syndrome.

## 2. Results

### 2.1. Identification and Quantification of CQA-Derivatives

**Figure 1:**
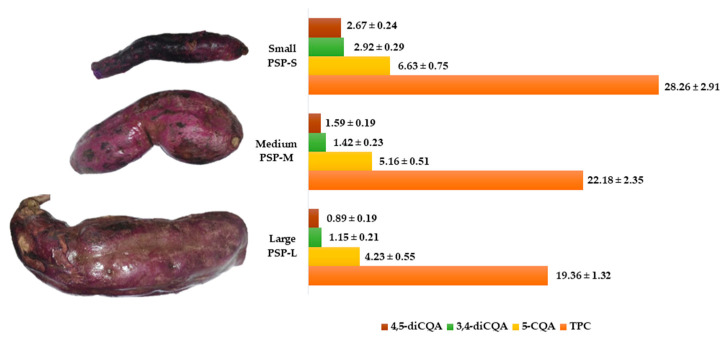
Total polyphenolic content (TPC), 5-CQA, 3,4-diCQA and 4,5-diCQA concentrations measured in 3 size of Mexican PSP: large (PSP-L), medium (PSP-M), and small (PSP-S). Each bar is the mean accompanied of the mean value ± SD of three independent experiments.

**Figure 2:**
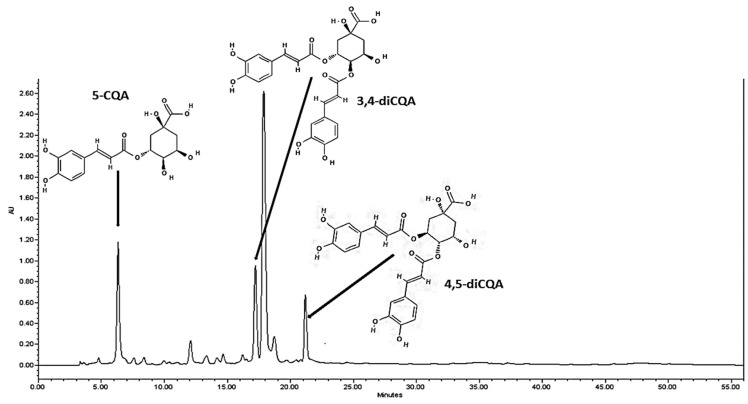
Chromatogram of CQA-derivatives from PSP and detected 320 nm.

Purple sweet potatoes were evaluated for total phenolic content (TPC) and the presence of CQA-derivatives according to the root size classified as PSP-L (>1 kg), PSP-M (0.2 ~ 1 kg), and PSP-S (<0.2 kg) as shown in Figure 1. TPC ranged from 19.36 to 28.26 mg/g DW among test tubers and it was the highest in PSP-S roots followed by PSP-M and PSP-L with TPC levels 27.4% and 46% lower than that found in PSP-S, respectively. Subsequently, CQA-derivatives were extracted from the PSP crude extract by means of hydrophobic affinity extraction and separated using silica gel column chromatography. Three CQA-derivatives, namely 5-CQA, 3,4-diCQA and 4,5-diCQA, were quantitatively analyzed, and characterized by HPLC-ESI-MS (Table 1 and Figure 2) as shown in Figure 2, the test CQA-derivatives accounted for 32.4%, 36.8% and 43.24% of TPC in large, medium and small-size PSP tuberous roots; respectively. Of the three structures, the most abundant polyphenol was 5-CQA followed by 3,4- and 4,5-diCQA.

### 2.2. Effect of 5-CQA, 3,4-diCQA or 4,5-diCQA on Mitochondrial Function on Primary Hepatocytes

**Figure 3:**
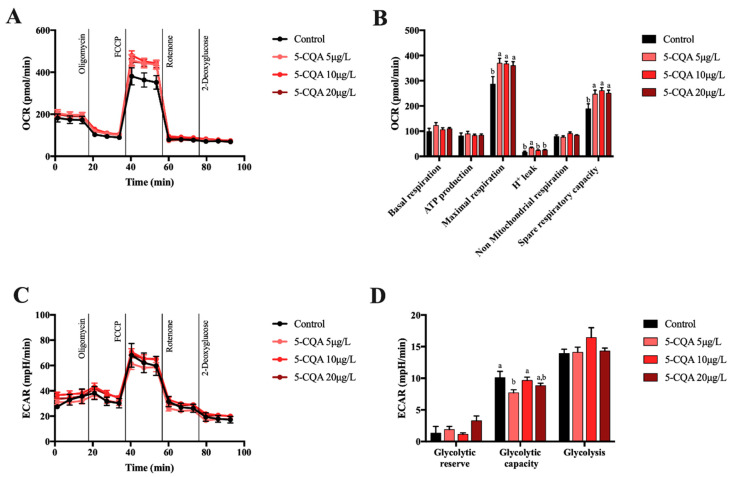
Oxygen consumption rate (OCR) (**A**), mitochondrial function parameters (**B**), extracellular acidification rate (ECAR) (**C**), and glycolytic parameters (**D**) in mouse primary hepatocytes incubated for 18 h with different concentrations of 5-CQA. Each bar represents the mean ± SEM of three independent experiments and analyzed by one-way ANOVA followed by Tukey multiple comparison post hoc test. The differences were considered statistically significant at *p* < 0.05. Mean values with different lowercase letters show statistical differences between each other.

To evaluate the effects of CQA-derivatives on mitochondrial function in mouse primary hepatocytes, mitochondrial stress tests were carried out using increasing concentrations of test compounds. It was observed that 5-CQA did not modify basal and non-mitochondrial respiration (Figure 3A,B) as well as oxygen consumption associated with ATP production. Interestingly, 5-CQA significantly increased the maximal respiration and spare respiratory capacity of primary hepatocytes which was indicative of the enhanced oxidative capacity in mitochondria (Figure 3B). However, this compound did not affect any glycolysis-related parameters, except for the glycolytic capacity that dropped in the presence of a 5 μg/L 5-CQA (Figure 3C,D). Overall, the obtained results indicated that 5-CQA had a potential to increase mitochondrial activity in primary hepatocytes.

**Figure 4:**
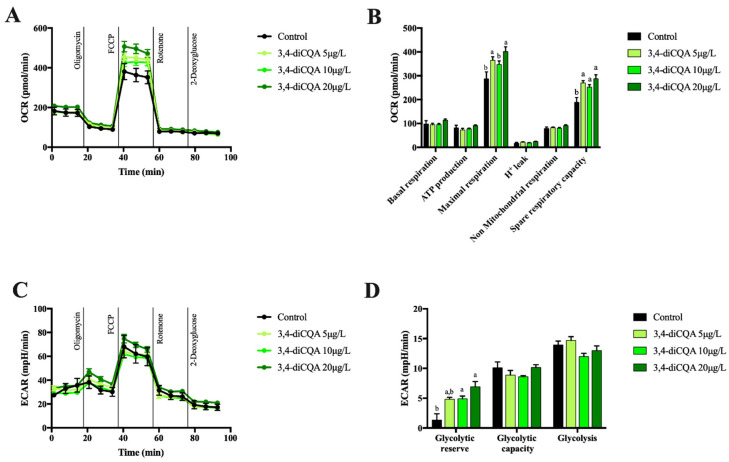
Oxygen consumption rate (OCR) (**A**), mitochondrial function parameters (**B**), extracellular acidification rate (ECAR) (**C**), and glycolytic parameters (**D**) in mouse primary hepatocytes incubated for 18 h with different concentrations of 3,4-diCQA. Each bar represents the mean ± SEM of three independent experiments and analyzed by one-way ANOVA followed by Tukey multiple comparison post hoc test. The differences were considered statistically significant at *p* < 0.05. Mean values with different lowercase letters show statistical differences between each other.

In case of 3,4-diCQA, OCR measurements showed no effect of this compound on basal and non-mitochondrial respiration as well as oxygen consumption associated with ATP production. However, values of the maximal respiration and spare respiratory capacity of primary hepatocytes significantly improved in the presence of 3,4-diCQA at all concentrations tested. The latter results demonstrated that mitochondrial oxidative capacity was enhanced in a similar manner to that exerted by 5-CQA (Figure 4A,B). Interestingly, 3,4-diCQA considerably increased glycolytic reserve (Figure 4C,D) which is a measure of cell capability to respond to an energy demand through glycolysis. In general, the data produced for 3,4-diCQA proved its positive effect on mitochondrial function in primary hepatocytes.

**Figure 5:**
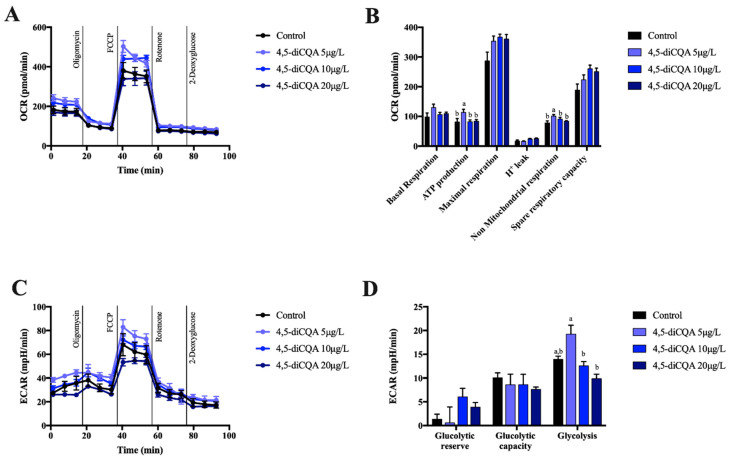
Oxygen consumption rate (OCR) (**A**), mitochondrial function parameters (**B**), extracellular acidification rate (ECAR) (**C**), and glycolytic parameters (**D**) in mouse primary hepatocytes incubated for 18 h with different concentrations of 4,5-diCQA. Each bar is the mean ± SEM of three independent experiments and analyzed by one-way ANOVA followed by Tukey multiple comparison post hoc test. The differences were considered statistically significant at *p* < 0.05. Mean values with different lowercase letters show statistical differences between each other.

The last CQA-derivative tested, namely 4,5-diCQA, was shown to have less effects on mitochondrial respiration and glycolysis than the other two compounds. Of the five mitochondrial parameters examined, it only affected oxygen consumption associated with ATP production and non-mitochondrial respiration at the lowest concentration tested (5 μg/L), as seen in Figure 5A,B. Similarly, 4,5-diCQA hardly modified glycolytic parameters, except for a slight increase of glycolysis at 5 μg/L (Figure 5C,D). In conclusion, the gathered results implied that 4,5-diCQA had little impact on mitochondrial function and could increase glycolysis at 5 μg/L concentration in primary hepatocytes.

### 2.3. Effect of 5-CQA, 3,4-diCQA or 4,5-diCQA on Fatty Acid Oxidation on Primary Hepatocytes

**Figure 6:**
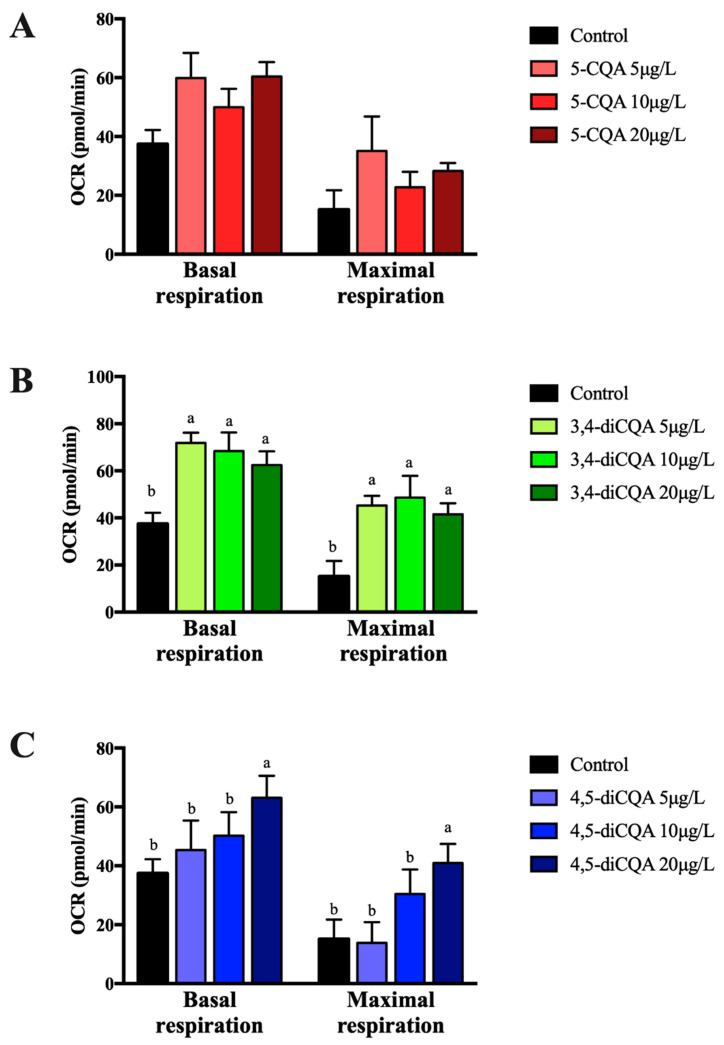
Oxygen consumption rate (OCR) using BSA-conjugated palmitate as a main substrate for oxidation to estimate fatty acid oxidation in mouse primary hepatocytes incubated for 18 h with the indicated concentrations of 5-CQA (**A**), 3,4-diCQA (**B**), and 4,5-diCQA (**C**). Each bar is the mean ± SEM of three independent experiments and analyzed by one-way ANOVA followed by Tukey multiple comparison post hoc test. The differences were considered statistically significant at *p* < 0.05. Mean values with different lowercase letters show statistical differences between each other.

To evaluate the effect of test CQA-derivatives on fatty acid oxidation in primary hepatocytes, mitochondrial stress tests were performed in presence of BSA-conjugated palmitate, as the only substrate for oxidation, and increasing concentration of each compound. The most pronounced effects were observed for 3,4-diCQA that enhanced fatty acid oxidation both under basal and maximal respiration conditions at all concentrations tested (Figure 6B). 5-CQA was demonstrated to affect fatty acid oxidation under basal respiration conditions, as can be seen in Figure 6A. 4,5-diCQA was effective only at 20 μg/L concentration for which a rise of fatty acid oxidation was observed under both experimental conditions (Figure 6C). Altogether, the obtained results indicated that CQA-derivatives had different capacity to increases fatty acid oxidation in hepatocytes.

## 3. Discussion

Purple sweet potatoes are known to be an excellent source of phenolic compounds that act as antioxidants. According to the root size classification applied in this work, PSP-L was found to have TPC levels comparable with those reported by Meng et al. [19] and Wang et al. [20] who determined the maximum phenolic concentration at 14.16 and 15.25 mg TPC/g DW (in S-3 variety), respectively. Interestingly, Mexican variety PSP-S not only had higher TPC than the values previously reported [21], but also it exceeded phenolic content of other edible roots and tubers, such as purple carrots (3.94 mg/g DW), Vitelotte-variety of purple potatoes (8.45 mg/g DW) [22], and P-3 variety of purple potatoes (10.02 mg TPC/g DW) [20]. 

As for CQA-derivatives content, our results indicated that their concentrations in PSP were higher than those found, for example, in purple-yellow carrot. Tiggiano carrots contained 5-CQA at 2.6 mg/g DW [23] while purple potatoes were reported to have 5-CQA at concentrations up to 12.5 mg/g DW [24]. Besides, the latter had low diversity of CQA-derivatives since approximately 90% of their phenolic content were represented by 5-CQA. Noteworthy, diverse structures of CQA-derivatives were present in PSP used in this study, and their concentrations were higher than that found in similar types of tubers. In consequence, the Mexican PSP-S variety seems to be a good source of TPC, in particular CQA-derivatives.

Since CQA-derivatives have been implied to play a role in many biological processes due to their antioxidant properties, our work focused on evaluation of their capacity to affect disturbed mitochondrial functions. This is especially relevant problem in a treatment of metabolic diseases such as obesity. Extracellular flux analysis allowed thorough measurements of mitochondrial oxidation parameters as well as cell glycolytic activity under basal and stressed conditions. We demonstrated that addition of 5-CQA or 3,4-diCQA increased the maximal respiration and the spare respiratory capacity in primary hepatocytes. Both parameters are indicative of the improved mitochondrial function. Another test compound, namely 3,4-diCQA, had a positive effect on hepatic capability to respond to an energy demand through glycolysis. On the other hand, 4,5-diCQA did not modify mitochondrial respiration and only increased glycolysis at the lowest concentration tested. Our data are consistent with those reported for CQA-derivatives and their metabolites extracted from chicory (*Cichorium intybus* L.), which were shown to promote increased mitochondrial respiration and cellular metabolism in rat hepatoma cells [25].

Based on these encouraging results of the current study, we propose the potential use of CQA-derivatives as auxiliary agents in the treatment of diseases involving mitochondrial dysfunction. The latter is caused partially by alterations in the electron transport chain, defects of electrical and chemical transmembrane potential, or reduction of critical metabolites [26].

The obtained data suggested that the presence of 5-CQA, and 3,4-diCQA could overcome disruptions in the electron transport chain, since it induced a higher increase on OCR with the use of FCCP, a potent uncoupling agent, that would be only possible with a functional electron transport chain. Besides, our results suggest that CQA-derivatives may not modify the function of the ATP synthase, also known as complex V, since CQA-derivatives did not modify the response in the presence of oligomycin, a well-known inhibitor of complex V, which remains to be evaluated in permeabilized and fully substrate-saturated cells. In addition, a limitation of our study, and a lack of information in the literature are regarding the moiety of CQA-derivatives, and the cellular proteins involved in the response. With this regard, Jackson et al. provided evidence that the caffeic acid moiety was important to maintain the effects on mitochondrial function [25].

As for proteins involved in the cellular response, we can speculate that AMPK may be implicated in this process. Zhang et al. [27] reported the activation of AMPK in the liver of hamsters following the addition of an extract rich in CQA-derivatives from *Pandanus tectorius*. Notably, AMPK modulates mitochondrial function at two levels. In the first place, AMPK activates peroxisome proliferator-activated receptor-γ co-activator 1α (PGC1-α), which in turn increases the activity of transcription factors involved in mitochondrial biogenesis [28]. Secondly, AMPK phosphorylates the mitochondrial fission factor (MFF) that regulates mitochondrial fission [29], i.e., a process that increases number of mitochondria. On the other hand, during stress insults such as nutrient deprivation, AMPK activation allows mitophagy of mitochondrial fragments without proper membrane potential [30]. Furthermore, other phenolic compound such as isoflavone genistein can activate AMPK leading to an increase in fatty acid oxidation [18]. Our results demonstrated that CQA-derivatives significantly increased hepatocytes capacity to oxidize fatty acids. We have previously observed that a rise in fatty acid oxidation improves hepatic steatosis in mice fed a high fat diet [31]. Therefore, further studies are required to fully address the role of CQA-derivatives in the improvement of this condition.

Due to their metabolic activity, mitochondria are the mayor source of reactive oxygen species (ROS) produced in the living cells. However, elevated levels of ROS can contribute to oxidative stress [32], which is the cause of lipid peroxidation, protein damage, and other pathological tissue changes associated with metabolic diseases, such as obesity, type 2 diabetes, as well as neurological disorders such as Alzheimer’s and Parkinson´s diseases [33,34]. Recently, Jiang at al. have observed the protective role of CQA against H_2_O_2_-induced injury in SH-SY5 neuroblast cells through a mechanism that involved activation of endogenous antioxidant enzymes [35]. Thus, it is possible that the observed improvements of mitochondrial performance in our study could be attributed to the antioxidant properties of CQA-derivatives. In fact, a caffeoylquinic acid-rich fraction (CQAF) of *Periploca forrestii* decreased malondialdehyde (MDA) levels, a reactive aldehyde produced by lipid peroxidation, in rats treated with 125 to 500 mg/kg of CQAF [36]. Moreover, moderate consumption of a soluble green/roasted coffee rich in caffeoylquinic acid decreased MDA levels in healthy and Hypercholesterolemic subjects [37], highlighting the therapeutic potential of CQA-derivatives.

Mitochondrial dysfunction plays a significant role in the development of non-alcoholic fatty liver disease (NAFLD) that includes hepatic steatosis, and non-alcoholic steatohepatitis (NASH), which can progress to fibrosis and cirrhosis [38]. NAFLD is a common comorbidity of obesity, type 2 diabetes and metabolic syndrome. In fact, 53% of obese children and 65% of adults suffering from class I and II obesity are diagnosed with NAFLD [39,40]. Therefore, it could be speculated that CQA-derivatives, that we proved they acted on primary hepatocytes, have a potential to be used as nutritional supplements in NAFLD treatment. This hypothesis can be supported by the results reported for animal model of diet-induced obesity. Huang et al. [11] demonstrated that 5-CQA contributed to a decrease of free fatty acids levels in hepatocytes of rats fed a high-fat diet through modulation of transcription factors, such as PPARα and liver X receptor α (LXRα), known for their involvement in lipid oxidation.

Finally, numerous studies have shown a scope of biological effects of CQA-derivatives extracts obtained from different sources, such as Asteraceae plants, blueberry, Kuding tea, Korean mountainous vegetables [41], to name a few. Among reported activities were inhibition of digestive enzymes [13], hypoglycemic effect through GLUT2 regulation in hepatic cells [12], reduction of body weight and blood lipids levels [42,43], modulation of lipid metabolism and intestinal microbiota in high-fat fed mice [44]. Our findings add to the list of biological roles played by CQA-derivatives in living cells, i.e., modulation of hepatic mitochondrial functions. Moreover, our in vitro study of hepatic cells is relevant to an organism as a whole, since absorption of CQA-derivatives by human organism has been already demonstrated [45]. Since each of the CQA-derivative tested in the present work demonstrated different effect on mitochondrial performance in a dose-dependent manner, it can be envisaged that the cumulative impact of several CQA-derivatives could enhance therapeutic effectiveness. Therefore, further research will focus on determining the dosage-effect relationship of a single CQA structure as well as a mixture of CQA-derivatives to achieve the desired biological response.

## 4. Materials and Methods 

### 4.1. Plant Purchase, Processing, and Extraction of Phenolic Content

PSP were obtained from a commercial sample acquired at the main wholesale market in Mexico City (Central de Abastos, CDMX, Mexico) from the second winter harvest. The PSP lot was classified according to the size: large (PSP-L) those that weighed more than 1.0 kg, medium (PSP-M) those that weighed in a range 0.2–1.0 kg, and small (PSP-S) those that weighed less than 0.20 kg. Each tuberous root was cut into 0.5 cm thick pieces that were stored in paper bags at −80 °C in an ultra-freezer ELT-13V-85 A 30 (Thermo Scientific Revco, Waltham, MA, USA) prior usage. PSP slices were lyophilized using FreeZone lyophilizer 4.5 (Labconco, Kansas City, MO, USA), pulverized and sieved through 60 mesh size. The powder was stored for further use. The phenolic compounds were extracted by sonication in a model 8892 ultrasonic cleaner (100 W, Cole-Parmer, Vernon Hills, IL, USA) at 40 °C for 20 min using 15 g of lyophilized PSP powder resuspended in 250 mL of solvent mixture consisting of acetone:water:lactic acid (40:60:1). The extract was then filtered through Whatman filter paper grade 4 (Sigma-Aldrich, St. Louis, MO, USA).

### 4.2. Quantification of Total Content of Phenolic Content

To determine the total phenolic content (TPC) in the samples, the method previously described by Taga et al. [46] was followed. In brief, 100 μL of the filtered extract were mixed with 2 mL of 2% Na_2_CO_3_ (*w*/*v*) and left to stand for 2 min. Following that time, 100 μL of Folin-Ciocalteu (Sigma-Aldrich, St. Louis, MO, USA) reagent diluted with H_2_O 1:1 was added and left to stand for 30 min. Next, the absorbance at 750 nm was measured using Cary 60 UV-Vis Agilent Technologies spectrophotometer (Agilent Technologies, Santa Clara, CA, USA). For calibration curve, a set of tannic acid standard (Sigma-Aldrich, St. Louis, MO, USA) solutions was prepared at concentrations ranging from 0.03 mg/mL to 1.0 mg/mL. The phenolic compound content was determined by comparison with the standard calibration curve. The results were expressed as mg of TPC/g of PSP per DW. All tests were performed in triplicate.

### 4.3. Separation and Purification of CQA-Derivatives

To purify 5-CQA, 3,4- and 4,5-diCQA, firstly, the crude extract was concentrated under reduced pressure on a Rotavapor (Buchi, Flawil, Switzerland); secondly, dissolved in 50 mL of H_2_O; thirdly, 150 mL of hexane was added, and the aqueous fraction was recovered; and finally, 150 mL of ethyl acetate was added, and the organic fraction (fraction enriched in CQA-derivatives) was recovered. Each of the CQA-derivatives was isolated by column chromatography using high-purity grade silica gel (Sigma-Aldrich, St. Louis, MO, USA) with a gradient elution of 50% hexane-methylene chloride, 100% methylene chloride, 50% methylene chloride-ethyl acetate, and 100% ethyl acetate. The characterization of the CQA-derivatives was carried out by HPLC-MS. The purity of the 5-CQA, 3,4- and 4,5-diCQA, was calculated by HPLC.

### 4.4. Identification of CQA-Derivatives

Identification of 5-CQA, 3,4- and 4,5-diCQA was determined using HPLC. Likewise, its presence was confirmed by an analysis of MS-HPLC, and they were compared with the retention time of commercial standards (5-CQA (Sigma-Aldrich, St. Louis, MO, USA), and 3,4- and 4,5-diCQA (Chengdu Biopurify Phytochemicals Lt, Chengdu, China). The HPLC analysis was performed on a Hypersil Gold C18 selectivity column 250 mm × 4.6 mm; 5 μm (Thermo Scientific, Waltham, MA, USA) using Waters system 1525 (Waters, Mildford, MA, USA) equipped with an autosampler Waters 2707 and dual UV-VIS detector Waters 2487 (320 nm). The binary mobile phase consisted of (A) water:acetonitrile:formic acid with at a volume ratio of 89:10:1 and (B) acetonitrile delivered at a flow rate of 1.0 mL/min. The gradient of 3–25% B in 45 min, 30% B in 2 min (at 47 min) and 3% B in 9 min (at 56 min) was applied.

The characterization of CQA-derivatives (Table 1) was carried out using HPLC-MS. An Agilent 6410 (Agilent Technologies, Santa Clara, CA, USA) Triple Quad LC/MS system was used, equipped with a G1311A binary pump, a G1316A thermostat column, and a G1367E auto-sampler with an electrospray ionization source (ESI). Separation was achieved using Hypersil-Gold column 250 mm × 4.6 mm, 5 μm (Thermo Scientific, Waltham, MA, USA) at 25 °C. The binary mobile phase consisted of (A) water:acetonitrile:formic acid at volume ratio of 89:10:1 and (B) acetonitrile delivered at a flow rate of 1.0 mL/min and gradient of 97–75% A in 45 min, 70% A in 1 min, and 97% A in 9 min. MS detection was performed using the negative ion mode, and spectra was registered for *m*/*z* between 200 and 2000. Other MS acquisition parameters were as follows: capillary voltage = 4000 V; nebulizer gas (nitrogen) = 50 psi; dry gas flow rate = 12 L/min; source heater dry gas temperature = 350 °C. The mass spectrometer was programmed to perform a full scan (MS), and zoom scans of the 353 and 515 *m*/*z* ions in the first scan (MS^2^). The ions were monitored with collision energy of 15 eV.

### 4.5. Quantification of 5-CQA, 3,4- and 4,5-diCQA

To prepare HPLC standard curves of each test compounds, 5-CQA, 3,4- and 4,5-diCQA were weighed out accurately and dissolved in 80% methanol (*v*/*v*) at concentration of 1 mg/mL. Standard solutions were prepared by a serial dilution of the stock solution in 80% methanol (*v*/*v*) to obtain the following concentrations: 0.20, 0.10, 0.05, 0.025 and 0.012 mg/mL. Standard curve equation and standard error of the regression corresponding to each CQA derivative were as follows:

y_5-CQA_ = 3E + 07x + 92229, R^2^ = 0.997 (1)

y_3,4-diCQA_ = 2E + 07x + 7745.2, R^2^ = 0.998 (2)

y_4-5-diCQA_ = 2E + 07x + 44069, R^2^ = 0.998 (3)

### 4.6. Primary Hepatocyte Cell Culture 

To set up cell culture of mouse primary hepatocytes, male C57BL/6 mice in the 8–12 weeks age range were obtained from the Experimental Research Department and Animal Care Facility at the National Institute of Medical Sciences and Nutrition Salvador Zubirán (INCMNSZ). The Animal Care and Ethics Committee of the INCMNSZ approved all the procedures (approval number CICUAL-FNU-2002-20-22-1). Mouse primary hepatocytes were collected by in situ perfusion of the liver according to the method by Berry and Friend [47]. The procedure consisted of cannulating and exsanguinating the liver in vivo followed by a continuous perfusion with collagenase. Then, liver was isolated and placed in Hanks balanced salt solution (HBSS) to disaggregate the tissue. Cell suspension was filtered through a 70 μM mesh. Next, filtered cells were washed twice with HBSS and finally re-suspended in M199 medium (Sigma Aldrich, St. Louis, MO, USA) supplemented with 10% FBS, 1 × antibiotic, 0.1% BSA, 1 nM insulin, 100 nM dexamethasone and 100 nM triiodothyronine. 

### 4.7. Mitochondria Function Evaluation

Primary hepatocytes were seeded into a XFe96 microplate at a density of 4000 cells/well. The medium was changed after 4 h to remove unattached cells. Cells were then incubated with 5-CQA, 3,4-diCQA, or 4,5-diCQA at a concentration of 5, 10 or 20 μg/L for 18 h. Mitochondrial function was evaluated by carrying out a mitochondrial stress test and using an extracellular flux analyzer XFe96 (Agilent Technologies, Santa Clara, CA, USA). Briefly, cells were washed and incubated for 1 h in a non-CO_2_ incubator with XF basal medium supplemented with 10 mM glucose, 1 mM pyruvate and 2 mM glutamine. For the experiment, 2 μM oligomycin, 1 μM FCCP, 1 μM rotenone/antimycin A and 50 mM of 2-deoxyglucose were sequentially injected, and three measurements were performed in basal conditions and after the addition of each compound. The experimental control were basal medium-treated cells. OCR and ECAR measurements were obtained and analyzed following the recommendations of the manufacturer. Basal mitochondrial respiration, ATP-linked respiration, proton leak, maximal respiration, non-mitochondrial respiration, and spare respiratory capacity were then calculated based on the OCR values. Glycolysis, glycolytic reserve, and glycolytic capacity were calculated based on the ECAR values as previously described [16]. Briefly, basal mitochondrial respiration is calculated by subtracting the average OCR after rotenone/antimycin A injection from the average basal OCR; ATP-linked respiration is calculated by subtracting the average OCR after oligomycin injection from the average basal OCR; proton leak is calculated by subtracting the average OCR after rotenone/antimycin A injection from the average OCR after oligomycin injection; maximal respiration is calculated by subtracting the average OCR after rotenone/antimycin A injection from the average OCR after FCCP injection; non-mitochondrial respiration is the average OCR after rotenone/antimycin A injection, and spare respiratory capacity is calculated by subtracting the average basal OCR from the average OCR after FCCP injection. Glycolysis was calculated by subtracting the average ECAR after 2-deoxyglucose injection from the average basal ECAR; glycolytic reserve was calculated by subtracting the average basal ECAR from the average ECAR after oligomycin injection, and glycolytic capacity was calculated by subtracting the average ECAR after 2-deoxyglucose injection from the average ECAR after rotenone/antimycin A injection. To evaluate fatty acid oxidation (FAO), the mitochondrial stress test was performed in basal medium containing minimal concentration of glucose (0.5 mM), and carnitine (0.5 mM) and BSA-conjugate palmitate (170 μM) as main substrate for oxidation. Basal and maximal respiration were calculated as described above. BSA-conjugated palmitate was prepared by dissolving sodium palmitate in a NaCl solution in a water bath at 60 °C, and the transferred to a BSA solution at 37 °C with constant agitation for 30 min to a final concentration of 1 mM of palmitate and 0.17 mM of BSA.

### 4.8. Statistical Analysis

The results are presented as mean ± standard error of the mean (SEM), all the experiments were performed in triplicate and analyzed by one-way ANOVA followed by Tukey multiple comparison post hoc test. The differences were considered statistically significant at *p* < 0.05 using GraphPad Prism 7.0 (GraphPad Software, San Diego, CA, USA).

## 5. Conclusions

The present study demonstrated the inverse relationship between the size of purple sweet potato and CQA-derivatives content, with the highest concentration of these compounds found in PSP-S. In addition, caffeoylquinic acid derivatives, namely 5-CQA and 3,4-diCQA, were shown to modulate mitochondrial activity while 3,4-diCQA and 4,5-diCQA increased the hepatic cells capacity to perform glycolysis in response to energy demand. Finally, our results indicate that the test CQA have a potential to improve impaired mitochondrial function in hepatocytes and to increase fatty acid oxidation. Such alternations in mitochondrial activity typically led to hepatic steatosis during obesity, diabetes, and metabolic syndrome.

## Figures and Tables

**Table 1:** CQA-derivatives in PSP identified by mass spectrometry (HPLC-MS).

*t_R_* (Min)	Compound	Molecular Formula	Molecular Ion (M-H) (*m*/*z*)	Fragments (*m*/*z*)	% of Purity
6.2	5-CQA	C_16_H_18_O_9_	353	191	97
17.7	3,4-diCQA	C_25_H_24_O_12_	515	353	95
21.5	4,5-diCQA	C_25_H_24_O_12_	515	353	96

## Data Availability

The data presented in this study are available on request from the corresponding author.

## Sample Availability

Samples of the compounds are not available from the authors.
